# Phospholipids Trigger *Cryptococcus neoformans* Capsular Enlargement during Interactions with Amoebae and Macrophages

**DOI:** 10.1371/journal.ppat.1002047

**Published:** 2011-05-26

**Authors:** Cara J. Chrisman, Patricia Albuquerque, Allan J. Guimaraes, Edward Nieves, Arturo Casadevall

**Affiliations:** 1 Department of Microbiology and Immunology, Albert Einstein College of Medicine, Bronx, New York, United States of America; 2 Department of Developmental and Molecular Biology, Albert Einstein College of Medicine, Bronx, New York, United States of America; 3 Department of Biochemistry, Albert Einstein College of Medicine, Bronx, New York, United States of America; 4 Department of Medicine, Albert Einstein College of Medicine, Bronx, New York, United States of America; University of Wisconsin-Madison, United States of America

## Abstract

A remarkable aspect of the interaction of *Cryptococcus neoformans* with mammalian hosts is a consistent increase in capsule volume. Given that many aspects of the interaction of *C. neoformans* with macrophages are also observed with amoebae, we hypothesized that the capsule enlargement phenomenon also had a protozoan parallel. Incubation of *C. neoformans* with *Acanthamoeba castellanii* resulted in *C. neoformans* capsular enlargement. The phenomenon required contact between fungal and protozoan cells but did not require amoeba viability. Analysis of amoebae extracts showed that the likely stimuli for capsule enlargement were protozoan polar lipids. Extracts from macrophages and mammalian serum also triggered cryptococcal capsular enlargement. *C. neoformans* capsule enlargement required expression of fungal phospholipase B, but not phospholipase C. Purified phospholipids, in particular, phosphatidylcholine, and derived molecules triggered capsular enlargement with the subsequent formation of giant cells. These results implicate phospholipids as a trigger for both *C. neoformans* capsule enlargement *in vivo* and exopolysaccharide production. The observation that the incubation of *C. neoformans* with phospholipids led to the formation of giant cells provides the means to generate these enigmatic cells *in vitro*. Protozoan- or mammalian-derived polar lipids could represent a danger signal for *C. neoformans* that triggers capsular enlargement as a non-specific defense mechanism against potential predatory cells. Hence, phospholipids are the first host-derived molecules identified to trigger capsular enlargement. The parallels apparent in the capsular response of *C. neoformans* to both amoebae and macrophages provide additional support for the notion that certain aspects of cryptococcal virulence emerged as a consequence of environmental interactions with other microorganisms such as protists.

## Author Summary

A key event in *C. neoformans* pathogenesis is capsule enlargement in mammalian hosts. Historically, this phenomenon was attributed to high CO_2_ and iron deprivation but the magnitude of capsular enlargement observed *in vivo* cannot be consistently replicated *in vitro*. This paper reports that *C. neoformans* responds to polar lipid extracts with massive capsule enlargement, with some cells having dimensions comparable to the giant cells observed *in vivo*. Phospholipids are identified in this paper as the inducers of capsule enlargement. Our work is important because this is the first host-derived molecule that has been identified as a stimulus of massive capsule enlargement thus providing a potential mechanism for the capsular enlargement observed *in vivo*. Furthermore, the fact that the signal is common to both macrophages and amoebae suggests that the capsule enlargement response to phospholipids is a mechanism for fungal sensing of phagocytic cell predators. This provides another example of a correspondence between a possible environmental signal and a mechanism of virulence.

## Introduction

Certain environmental microbes exist that have no obvious need for animal virulence with regards to their survival or propagation yet these organisms have the ability to cause infection and disease in a human host. One such organism is the soil fungus *Cryptococcus neoformans*, a major pathogen for immunocompromised individuals, such as those with advanced HIV infection. *C. neoformans* has several well-characterized virulence factors [1], and the most extensively studied virulence factor is its polysaccharide capsule [2], [3]. The capsule is believed to contribute to virulence through multiple mechanisms as it is both anti-phagocytic and capable of causing detrimental effects on host immune system functions [3]. The polysaccharide capsule is also a powerful free radical sink that protects the fungal cell from oxidants, such as those produced in the oxidative burst of phagocytic cells [4]. A remarkable property of the capsule is its ability to undergo enlargement during infection and this phenomenon is associated with cryptococcal virulence in the mammalian host [5]. This enlargement can result in gigantic cells that exceed the size of macrophages [6], [7]. Several factors have been shown to induce this capsular enlargement, including high CO_2_, low iron, basic pH, and mammalian serum [8]. Additionally, capsular enlargement intensifies protection against both phagocytosis and oxidative damage [4], [9].

*C. neoformans* is a facultative intracellular pathogen with a unique replication strategy in macrophages [10], [11]. The sophisticated virulence strategies utilized by *C. neoformans* in the human host and the ability of cryptococcal polysaccharide to interfere with the immune response might suggest that such virulence factors as the polysaccharide capsule have evolved for evading mammalian defenses. However, given that *C. neoformans* does not require a mammalian host for replication and survival, the evolutionary origin of such sophisticated virulence strategies has been a perplexing problem in the field. Consequently, there has been considerable interest in characterizing the interactions of *C. neoformans* with other soil organisms. *Acanthamoeba polyphaga* was shown by Bunting *et al.* to interact with and ingest cryptococcal cells in classic studies carried out in the 1970s [12]. In 2001, Steenbergen *et al.* demonstrated that the interaction of *A. castellanii* with *C. neoformans* was similar to that observed with macrophages [13]. Recently, this concept has been extended to the emergence of fungal virulence for insects [14]. Our group also described the interaction of *C. neoformans* with three *Paramecium spp*., which were shown to rapidly ingest and kill the fungus [15]. These results led to the proposal that the virulence strategies used by *C. neoformans* for survival in mammalian hosts had emerged and developed through environmental interactions, due to the constant selection by predation [16], [17]. In this scenario, cryptococcal virulence factors are the result of environmental selection and serve this microbe in mammalian infection by the accidental adaptation to the host [18]. Additional evidence for this theory comes from the finding that non-lytic exocytosis from macrophages [19], [20] is also observed with amoeba [21].

Consequently, we hypothesized that the increase in capsule size may also occur in interactions with amoebae, perhaps as a mechanism to avoid phagocytosis by those predators. Upon co-incubation of the amoeba and the fungus, we observed that *C. neoformans* responded to the protist by increasing its capsule size. We have characterized the properties of capsule–inducing amoebic extracts, including their composition, stability, and effects on the *C. neoformans* cells. Additionally, we have observed that the same response could be elicited by both live and dead macrophages. These observations led to a search for the signal sensed by *C. neoformans* and we report that phospholipids can trigger both capsule enlargement and giant cell formation.

## Results

### *C. neoformans* responds to *A. castellanii* with capsular enlargement

**Figure 1:**
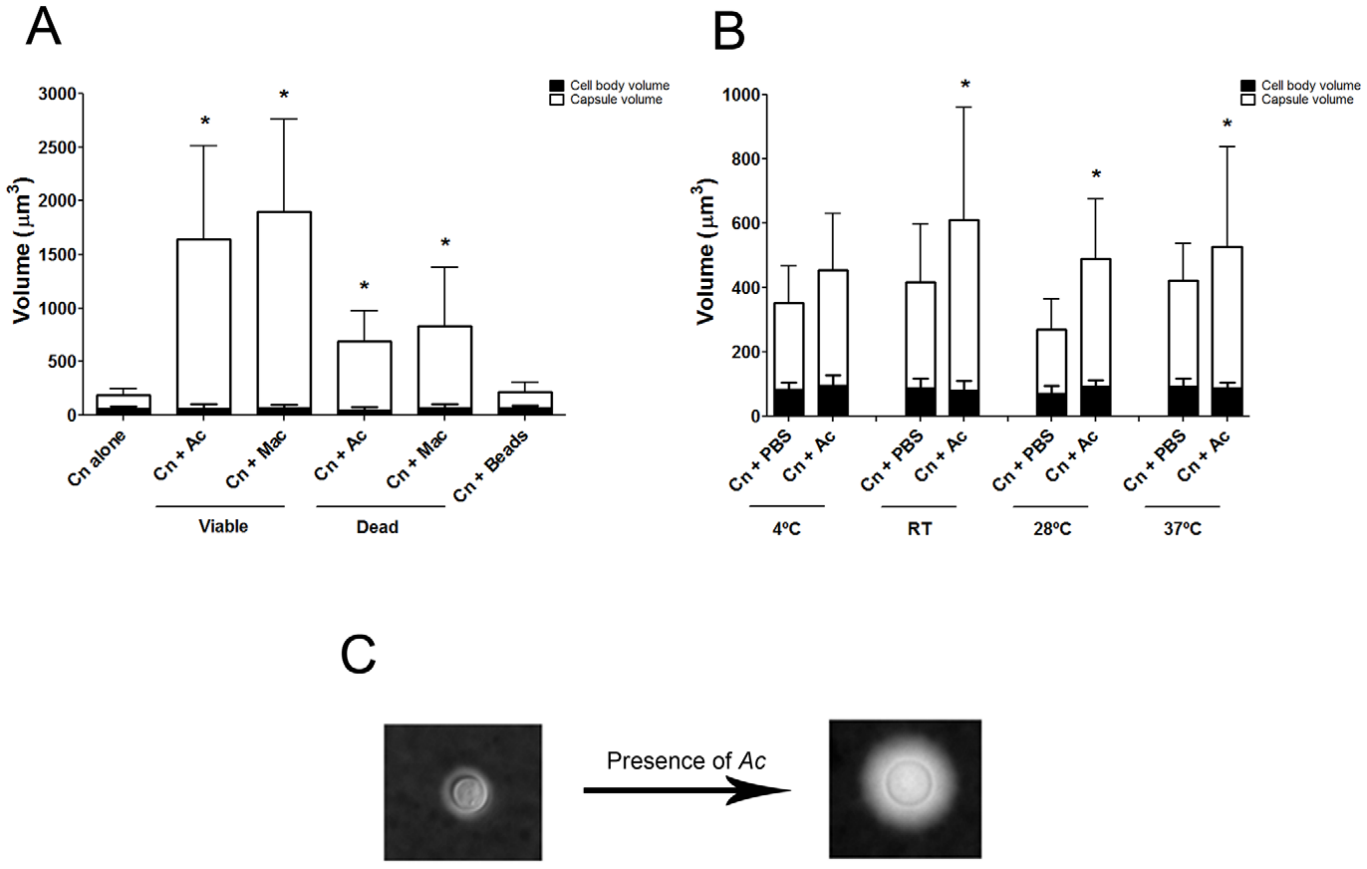
Capsule enlargement can be elicited by mammalian and protozoan cells regardless of viability, but not by mechanical stimulus. A) The *C. neoformans* (Cn) cells were incubated alone in PBS, with live and heat-killed *A. castellanii* (Ac), live and heat-killed J774.14 macrophage-like cells (Mac), and polystyrene beads. Live amoebae and macrophages were more effective than heat-killed cells in triggering capsular enlargement. In contrast, there was no enlargement in the presence of the polystyrene beads, when compared to incubation in PBS alone. Each assay was performed independently on 3 separate days and the numbers shown represent the average of a minimum of 50 *C. neoformans* cells. B) Capsule enlargement activity of *A. castellanii* extracts as a function of temperature. Activity assays were done at 4°C, room temperature (RT), 28°C, and 37°C. This experiment was done two times on separate days and each condition represents a minimum of 50 cells. C) Representative *C. neoformans* cells suspended in India ink showing enlargement of the polysaccharide capsule after incubation with *A. castellani* (40x). (*) indicates P<0.001 for capsule enlargement for each condition when compared to control incubation alone in PBS.

Co-incubation of *C. neoformans* with amoeba elicited an approximately four-fold increase in the cryptococcal capsular volume as compared with yeast cells in the absence of amoeba (Figure 1A and 1C, *p<0.0001). Increase in cryptococcal capsular volume was similarly observed when *C. neoformans* cells were incubated with macrophages as previously observed [22] (Figure 1A, *p<0.05). Increasing the incubation time to 48 h produced similar results (data not shown). Co-incubation of *C. neoformans* with either dead protozoa or macrophages also resulted in capsular enlargement, indicating that phagocytic cell viability was not required for this effect (Figure 1A), although both viable amoeba and macrophages induced a more pronounced effect, twice as much as the capsule increase observed with dead organisms.

### Capsular enlargement required cellular contact with amoebae

We investigated whether the capsule inducing molecule was diffusible by using 24-well flat-bottom plates where *C. neoformans* cells were separated from *A. castellanii* by 0.4 µm cell culture inserts which prevented the passage of either organism, but allowed the passage of small soluble molecules. The *C. neoformans* cells to be assessed were placed in PBS below the inserts to allow diffusion to carry any molecules to these cells. The conditions above the inserts were varied, and included PBS alone as a control, *A. castellanii* to determine if *A. castellanii* alone produced a stimulant molecule, and a combination of *A. castellanii* and *C. neoformans* to determine if the combination was necessary to stimulate production of a small molecule or its chemical modification. The *C. neoformans* capsules were measured at 24 and 48 hrs. Measurements included conditions in which only PBS was placed below the insert for 24 hrs and then *C. neoformans* was added for an additional 24 hrs in the event that the reaction required more time because of the physical separation, but no consistent effects on capsule enlargement were observed (data not shown). Additionally, when *C. neoformans* was placed in *A. castellanii* cell-free supernatants, no capsular enlargement was observed, suggesting that *A. castellanii* does not secrete a capsule-inducing molecule in solution (data not shown). We then set up a large volume co-incubation of *A. castellanii* and *C. neoformans* and tested the concentrated supernatant from this interaction in the activity assay. No activity was found, suggesting that the active moiety was either not released into the supernatant, that it remained on the surface of the amoeba, or that it rapidly lost activity in solution (data not shown).

We considered whether capsular enlargement was a result of mechanical stimulation by a foreign object such as the amoebae. This was tested by incubation of fungal cells with 9.2 µm polystyrene beads. There was no statistical difference between the capsule volume after incubation with beads and the volume when *C. neoformans* was incubated alone in PBS (Figure 1A, p>0.05), despite documenting that yeast and bead cells could be found in close proximity to one another (data not shown).

### Capsule enlargement occurred at a range of temperatures

Comparisons of the *C. neoformans* volume alone in PBS and after incubation with *A. castellanii* revealed that the capsule volume was enlarged at room temperature and above, specifically at 28°C and 37°C (Figure 1B, *p<0.05). The highest ratio of capsule induction was obtained with incubations at 28°C, which is the optimum temperature for the growth of the amoeba.

### Capsule enlarging compound(s) fractionate to the upper aqueous phase fraction following lipid extraction

**Figure 2:**
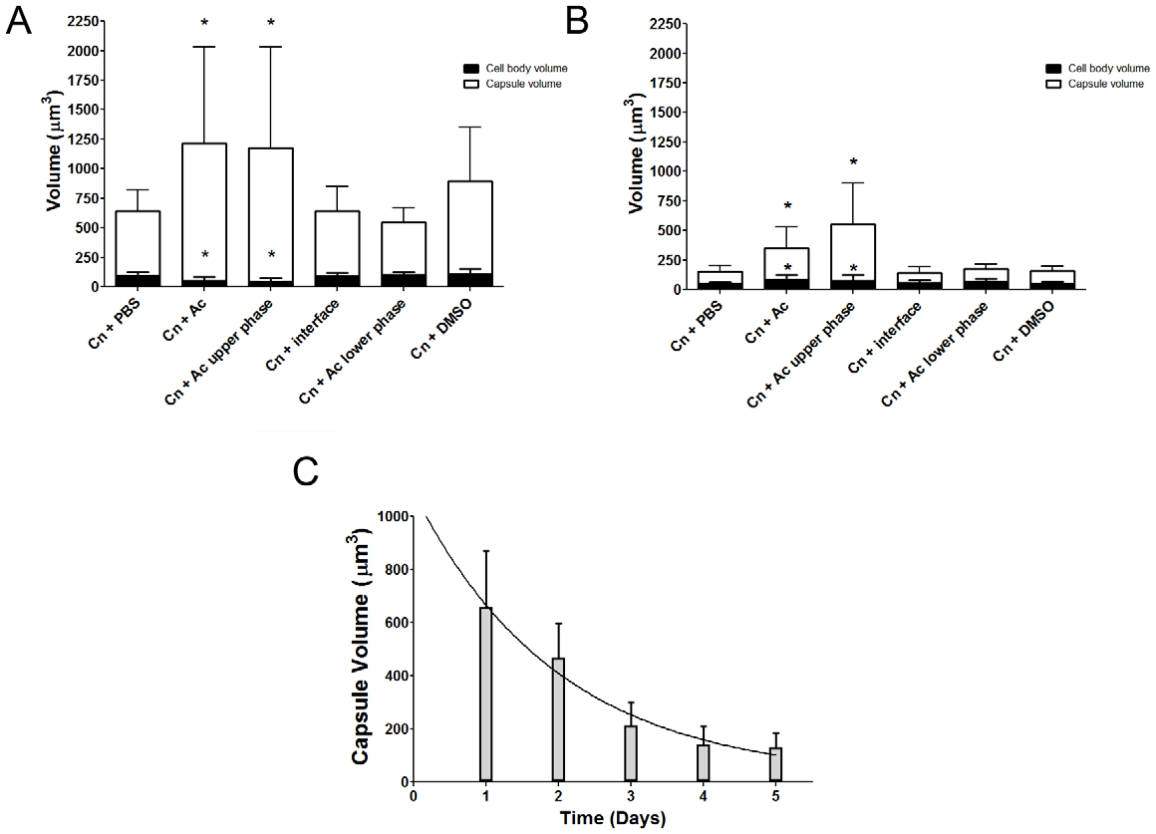
Polar fractions from *A. castellanii* extracts induce *C. neoformans* capsule enlargement. A) *C. neoformans* strain 24067 (Cn) was incubated alone, with intact *A. castellanii* (Ac), and with the three fractions (upper, interface, and lower) that resulted from lipid extraction of *A. castellanii.* The statistically significant increase in capsule volume was only observed with intact amoeba and with the upper phase polar fraction. Each condition represents a minimum of 50 *C. neoformans* cells and experiments were done in triplicate. P<0.05. B) *C. neoformans* strain H99 (Cn) was incubated in the same manner as 24067, with the same conditions as stated above. The effects for strain 24067 were proportionately greater than for strain H99. C) Capsule-inducing activity of *A. castellanii* extracts declines as a function of time. The half-life of activity decay at room temperature was 1.385 d.

In considering common triggers found in both macrophages and amoebae, we focused on membrane lipids, given that cell membranes are highly conserved in eukaryotic evolution. *A. castellanii* lipid extraction was performed using the Folch Method [23]. This procedure gave rise to three fractions (aqueous upper phase, interface, and organic lower phase) which were separated and incubated individually with *C. neoformans* serotype D strain 24067. After co-incubation with the extracted fractions for 24 and 48 h, we observed that only the upper polar phase, normally considered the “non-lipid” fraction, induced a significant increase in capsular volume that was comparable to that observed with intact *A. castellanii* (Figure 2A, p>0.05), while no effect was observed with the lower organic phase or the interface fractions. This effect of the upper polar phase on capsule enlargement was also observed using a *C. neoformans* serotype A strain, H99 (Figure 2B), with a more pronounced increase compared to controls, despite the lower absolute numbers compared to the serotype D 24067 strain. Additionally, when J774.14 macrophage-like cells were subjected to the same lipid extraction protocol and tested in the activity assay, we observed parallel results. Upon co-incubation with *C. neoformans,* the macrophage upper polar phase was again found to cause capsular enlargement equivalent to the intact cell (data not shown).

### *C. neoformans* growth in the presence of upper phase polar extract

Given that capsule enlargement has been linked to stationary cell growth [8], we investigated the effect of the *A. castellanii* polar extract on fungal growth. Using *C. neoformans* strain 24067, we compared the growth in PBS and in SDB for 24067 incubated either alone or with various concentrations of polar extract. In SDB, the growth curves were found to be identical. In PBS, growth rates were much slower, however, after 40 h, cells grown with lipid extract manifested increases in growth rate relative to PBS, presumably as a result of the fact that the extract could provide nutrients (data not shown).

### Stability of the amoeba extract

We noted that amoeba polar extracts often lost their ability to induce capsule growth upon storage or additional purification. Analysis of the stability of the capsule-inducing component over time revealed a rapid decrease of activity (Figure 2C) such that the half life of activity decay was calculated to be 1.385 days.

### Amoeba extracts induce the production of capsular polysaccharide and exopolysaccharide

**Figure 3:**
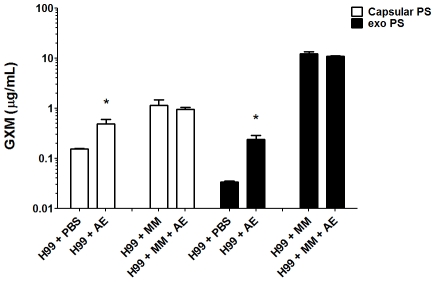
Polar fractions from *A. castellanii* extracts (AE) induce *C. neoformans* strain H99 exopolysaccharide release. This effect was apparent only when the experiment was carried out in PBS and no effect was observed when the yeast cells were suspended in minimal medium (MM), possibly due to glucose repression.

We evaluated the production of both capsular and exopolysaccharide following overnight incubation with amoeba extract in PBS or in minimal medium. Incubation of *C. neoformans* with the amoeba upper phase polar extract in PBS resulted in a 2-fold increase in capsular polysaccharide and a 6-fold increase in exopolysaccharide relative to the amount produced in PBS alone (Figure 3). When *C. neoformans* was incubated in minimum medium containing high glucose concentrations, no difference was observed for either capsular and exopolysaccharide (p>0.05) production, whether incubated with amoeba extract or alone in the medium.

### Requirement for phospholipase B

**Figure 4:**
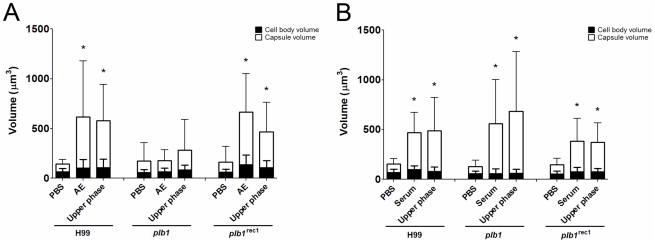
Phospholipase B mutants respond to serum components with an increase in capsule size but not to *A. castellanii*. A) *C. neoformans* strains H99, *plb1*, and *plb1* ^REC1^ were used in an activity assay with intact *A. castellanii* and the upper phase polar fraction. The wild type and reconstituted strains showed significant increases in capsule volume in the presence of the intact organism and the polar lipid fraction. The *plb1* mutant, however, did not show a statistically significant increase in either condition. The numbers shown represent the average of a minimum of 50 *C. neoformans* cells. The experiment was done independently on 3 separate days and the results were reproducible. B) The Fetal Calf Serum (FCS) upper phase fraction elicits capsular enlargement, but is not dependent on PLB. FCS was subjected to lipid extraction and separated into fractions. The fractions were tested in the activity assay with the *C. neoformans* strains H99, *plb1*, and *plb1* ^REC1^. The active component separated into the polar fraction, similar to results seen with intact *A. castellanii*; however, the effect was not dependent on PLB activity as the enlargement of the capsule was observed in the PLB-deficient strain. (*) indicates P<0.05 for capsule enlargement for each condition when compared to control incubation alone in PBS for each strain.

Since the capsule enlargement phenomenon required contact between *C. neoformans* and amoeba cells, we considered whether the release of polar lipids from amoeba membranes could be a step in amoeba-mediated capsular enlargement and thus evaluated the requirement for fungal phospholipase in this process. Phospholipase B (PLB) can release both *sn*-1 and *sn*-2 fatty acids from phospholipids [24]. *C. neoformans* produces extracellular PLB and both PLB-deficient (*plb1*) and reconstituted (*plb1* ^REC1^) strains have been generated on an H99 background [24]. Both the parental and the reconstituted strains exhibited the same increase in capsule volume when incubated with either intact *A. castellanii* cells or with the upper phase fraction, but not when incubated with PBS (Figure 4A, *p<0.05). However, the *plb1* mutant strain was unable to increase the capsular volume under any of these conditions, indicating a necessity for PLB production.

The necessity for PLB activity led us to investigate whether other phospholipases may also play a role in the interaction between the two organisms. The *C. neoformans* strain 24067, as well as the parental (H99), the phospholipase C mutant (Ä*isc1*), and the reconstituted (Δ*isc1^REC^*) strains were tested in the activity assays with *A. castellanii*. The *C. neoformans* phospholipase C mutant strain did not display any defects in capsule enlargement upon co-incubation with *A. castellanii* (data not shown).

### Serum upper phase lipid extract elicited enlargement in wild type and *plb1* strains

The *C. neoformans* capsule can be enlarged by incubation in 10% fetal calf serum (FCS), as described [22]. Consequently, we investigated whether the capsule-inducing properties of serum were also due to polar lipids and whether the effect was also PLB-dependent. Using the same extraction protocol that was used with *A. castellanii*, FCS was separated into upper phase, interface, and lower phase fractions. *C. neoformans* strains H99, *plb1*, and *plb1* ^REC1^ were tested in activity assays where they were each incubated with the FCS fractions (Figure 4B). Similar to the results with amoebae, the FCS capsule-inducing activity was also found in the upper polar fraction (p<0.05). However, unlike the *A. castellanii* polar extract, the FCS polar lipid fraction induced enlargement of the *plb1* capsule, suggesting that for serum-derived polar lipids, there is no PLB requirement for capsular enlargement.

### Effect of heat, glucanase, and protease on extract activity

**Figure 5:**
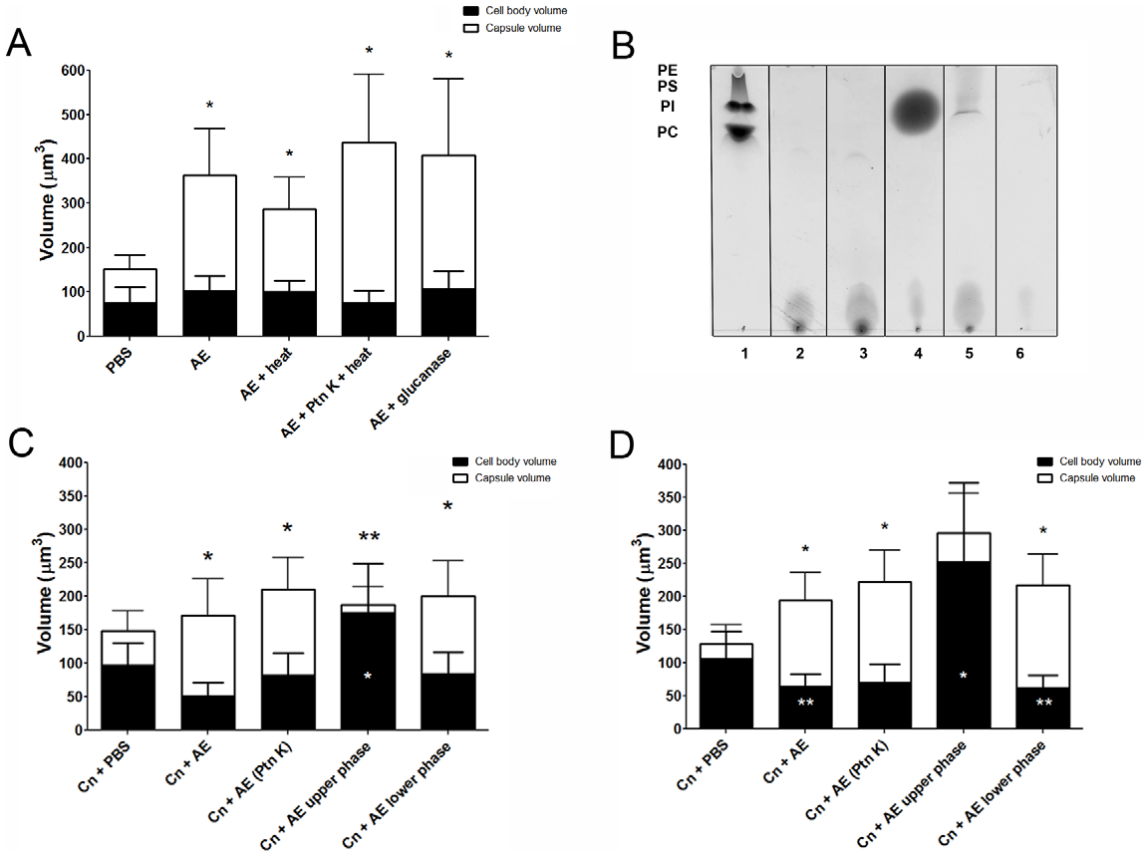
Capsular enlargement in response to different extracts. A) Activity of upper phase amoebae extracts (AE) on *C. neoformans* strain H99 after extract treatment with heat, Proteinase K (Ptn K), and glucanases. Neither heat nor glucanase treatments had a significant effect on capsule enlargement activity. In contrast, Proteinase K + heat treatment elicited cells with larger capsule volumes. This experiment was done two times on separate days and the results were reproducible. The numbers shown represent the average of a minimum of 50 *C. neoformans* cells. B) Thin liquid chromatography shows an increase in the mobility of the phospholipid bands upon treatment of amoebae extracts with Proteinase K, and fractionation of the free lipid compounds to the lower phase fraction. Lane 1: Phospholipid markers: (PE) phosphatidylethanolamine; (PS) phosphatidylserine; (PI) phosphatidylinositol; and (PC) phosphatidylcholine; Lane 2: Untreated extracts; Lane 3: Untreated extract + Proteinase K (before treatment); Lane 4: Proteinase K-treated extract; Lane 5: Lower phase fraction after treatment with Proteinase K; Lane 6: Upper phase fraction after treatment with Proteinase K. C) and D) Demonstration that the active substance in the amoeba extract (AE) shifts from the upper phase to the lower phase after digestion with Proteinase K (Ptn K). Panels C and D show results from overnight and 48 h, respectively. Chloroform extraction of the upper phase after Proteinase K digestion (Cn + AE upper phase) enhances the activity of the upper phase to promote cell body size increase, while the capsule inducing substance transfers to the lower phase (Cn + AE lower phase). (*) indicates P<0.05 for capsule enlargement for each condition when compared to control incubation in PBS. In Panel C, the (**) indicates P<0.05 for a decrease in capsule relative to incubation in PBS, while in Panel D (**), indicates P<0.05 for a decrease in cell body volume relative to incubation in PBS.

Both heat and glucanase treatments were found not to affect the ability of the extract to trigger capsule enlargement (Figure 5A, p>0.05). However, when the fractions were treated with Proteinase K and heat, we observed a dramatic 39% increase in the capsule induction activity when compared to the untreated extract, suggesting that the active compound may be complexed with a protein in solution and that its cleavage helps to release the active compound. The fraction treated with Proteinase K and heat was subsequently run on a silica TLC plate with a mobile phase of a 65∶25∶4 ratio of methanol∶chloroform∶water (Figure 5B). A new band appeared, with a higher Rf, most likely consisting of free lipids released after proteinase digestion. We performed a new fractionation using the Folch method following the Proteinase K digestion. New upper and lower phases incubated overnight or for 48 h with *C. neoformans* showed different results. Activity was fractionated to the lower phase, which induced capsule enlargement compared to PBS, with similar levels to treatment with Proteinase K without fractionation, after both overnight (Figure 5C, *p<0.05) and 48 h (Figure 5D, *p<0.05) incubations. Interestingly, at both time-points evaluated, the new upper phase material obtained after fractionation of the Proteinase K digestion resulted in an increase in cell body volume when compared to PBS.

### Purified phospholipids and their polar heads induced capsule enlargement

The requirement of PLB for capsular enlargement combined with the new band observed in the polar extract TLC after Proteinase K treatment suggested that the enlargement activity was due to a type of phospholipid or phospholipid-derived molecule from the *A. castellanii* extracts. Thus, we tested the ability of a few purified commercially available lipids and lipid-derived molecules to trigger capsule enlargement.

**Figure 6:**
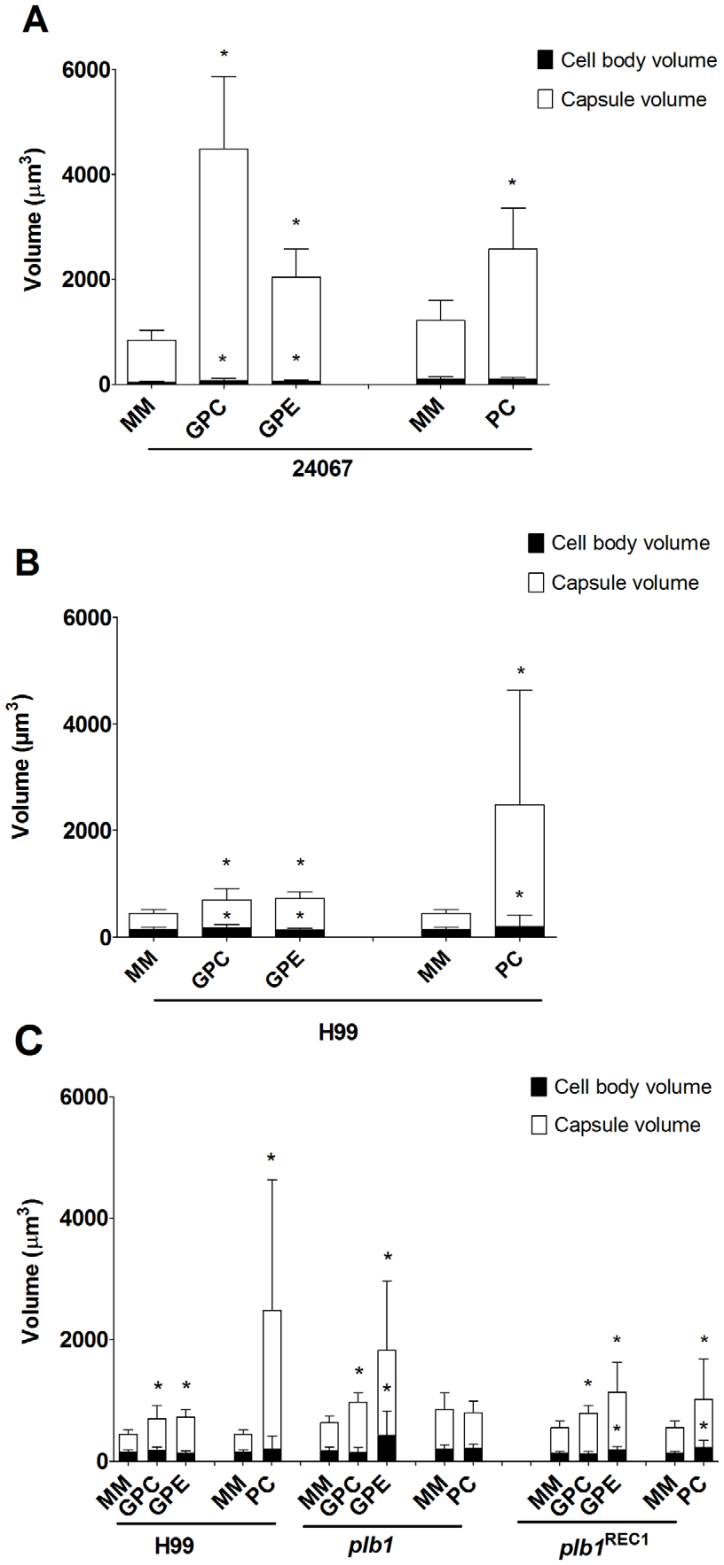
Capsule enlargement was elicited by glycerophosphocholine (GPC), glycerophosphoethanolamine (GPE), and phosphatidylcholine (PC). Activity assays were performed to test the effects of GPC, GPE, or PC on the capsule of *C. neoformans*. A) H99 and B) 24067 fungal cells were incubated alone in minimal medium (MM), or with 10 µM of GPC or GPE or 5 mM of PC for 48 hours. Each assay represents a minimum of 50 *C. neoformans* cells. C) H99, *plb1*,1 and *plb1* ^REC1^ cells were incubated either alone in MM or with 10 µM of GPC or GPE or 5 mM of PC for 48 hours. The numbers shown represents the average of a minimum of 50 *C. neoformans* cells. (*) indicates P<0.01 for capsule enlargement for each condition when compared to control incubation alone in MM.

One of those molecules was phosphatidylcholine (PC), a major component of amoeba cell membranes. We observed that PC was able to induce a dose-dependent capsule enlargement in two different strains of *C. neoformans* comparable to the one obtained in the co-incubation experiments (Figure 6A and 6B; Figure S1B). The average increase varied from 2- to 8-fold (p<0.001) depending on the conditions of the experiment, with larger increases when the cells were incubated in MM instead of PBS and for at least 48 hours. In addition to PC we also tested phosphatidic acid (PA), phosphatidylethanolamine (PE), phosphatidylglycerol (PG), phosphatidylinositol (PI), phosphatidylserine (PS) and lysophosphatidylcholine (LC) (Figure S1B). All the compounds with the exception of PG and PS produced significant enlargement of the C. *neoformans* capsule, however the effects were highest in the presence of either PC or LC.

Additionally, mass spectrometry of samples from the amoebae polar extract treated with or without Proteinase K, demonstrated the presence of phospholipids. However, phospholipid concentration was very low when compared to the amount detected in the untreated non-polar samples by mass spectrometry (data not shown), therefore precluding molecular identification.

*C. neoformans* PLB activity has been linked to the generation of arachidonic acid from fungal phospholipids and to the subsequent production of eicosanoids, including prostaglandins [25]. Thus, we hypothesized that arachidonic acid or one of its products could be responsible for the capsular enlargement, but none of the compounds tested promoted capsule growth (Table S1).

In addition, we considered that the effect on the *C. neoformans* capsule could be caused by the polar head group of the phospholipids, which also can be derived from PLB activity. It has previously been shown that GPC is the only degradation product of PC upon treatment with *C. neoformans* supernatants containing PLB activity [26]. Thus, two commercially available polar head groups, GPE and GPC, were tested with *C. neoformans* strains 24067 and H99 in the activity assay (Figure 6A and 6B, respectively). We observed that both molecules were able to induce capsule enlargement with differences in their effect dependent on the *C. neoformans* strain used (Figure 6A and 6B). After 24 hours of treatment, 10 µM of GPC was able to induce an average 2-fold increase in the capsule volume of 24067 and H99 cells (data not shown) reaching a 5-fold increase in the first strain after 48 hours of treatment. GPE produced an average 2-fold increase with both strains, however, was slightly but significantly more active with H99 cells. Additionally, GPE and GPC were able to induce capsule enlargement in the PLB-deficient strain comparable to the enlargement previously observed in the presence of amoeba and polar extracts (Figure 6C). In this case, GPE was also shown to be more active against the *plb1* mutant than GPC and was also able to induce the presence of giant-like cells in the mutant cultures. As observed with the polar extracts, both GPC and GPE were also shown to lose their activity very rapidly when in solution. We tested concentrations of GPC and GPE up to 1 mM, however, concentrations higher than 10 uM did not result in further increases in the capsule enlargement (Figure S2). Conversely, incubation of *plb1* mutant cells with intact phospholipids, such as PC, was not able to induce capsule enlargement, thus supporting the necessity of the phospholipase B activity in this process (data not shown).

*C. neoformans* PLB contains three enzyme activities in one protein, phospholipase B (PLB), lysophospholipase (LPL) and lysophospholipase transacylase (LPTA). These activities have been found to be either secreted or cell associated (either membrane bound or in the cytosol) [27]. PLB removes both acyl chains from phospholipids; LPL removes the single acyl chain from lysophospholipids; and LPTA adds an acyl chain to lysophospholipids to produce phospholipids. To further evaluate the role of PLB in the capsule enlargement, we tested the effects of three phospholipase inhibitors (as described [27]) on the capsule enlargement induced by PC. The first inhibitor was alexidine dihydrochloride (compound AX) which primarily inhibits secreted PLB activity at the tested concentration. Another inhibitor was dioctadecyldimethylammonium bromide (compound O) which acts mainly on secretory and cytosolic LPL and LPTA and on cell-associated PLB. The third inhibitor was palmitoyl carnitine (compound PAC), which has been found to be a potent inhibitor of PLB activity at 0.5 mM while affecting LPL and LPTA activities by only by 35% [28]. We found that compound AX did not affect the capsule enlargement induced by PC, however both compound O and compound PAC, which target cell-associated PLB and secreted and cell associated LPL and LPTA activities, abolished the capsule enlargement (Figure S3). These results further support the role of phospholipase B in the capsule enlargement and suggest that the PLB activity involved in the process is possibly cell-associated.

### Prolonged incubation times resulted in induction of giant cells

**Figure 7:**
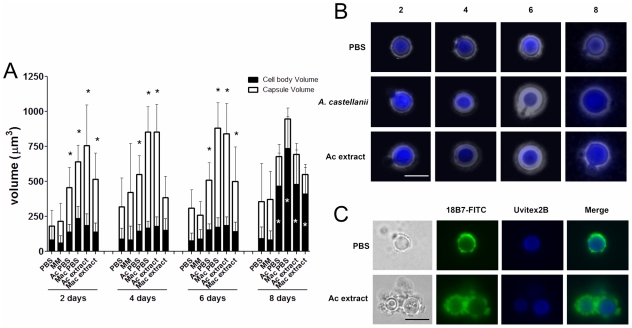
*C. neoformans* capsule and cell size growth in response to *A. castellani* (Ac), macrophages (Mac), and their upper phase extracts as a function of time. A) Initial effects involve capsule enlargement but continued incubation results in cells with giant cell bodies. B) Uvitex 2B preparations demonstrate an increased double-layered capsule of *C. neoformans* over time when co-incubated with *A. castellanii* cells and extracts. An increased cell body size, along with a decreased capsule size, was observed after an 8 day incubation period. C) Immunofluorescence demonstrates that *C. neoformans* cells are more intensely labeled with capsular antibodies following treatment with *A. castellanii* cells and extracts. Scale bars  = 10 µm.

**Figure 8:**
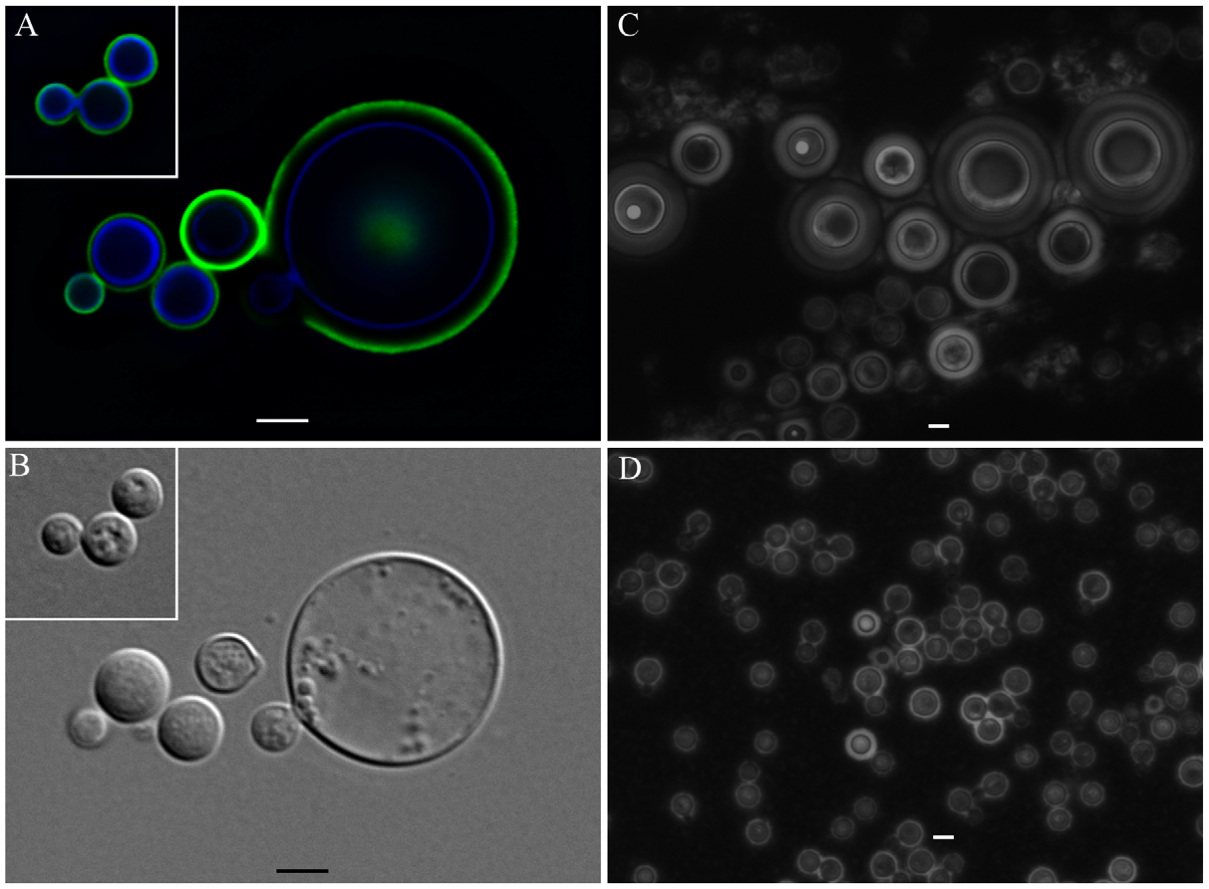
*C. neoformans* cell gigantism following treatment with phosphatidylcholine (PC). A) Deconvolved immunofluorescence of H99 cells treated with 5 mM PC for 48 hours and labeled with Uvitex 2B (blue) and DTAF-18B7 (green). Inset represents untreated cells under the same magnification. B) Differential interference contrast of Panel A. C) India ink preparation of H99 cells treated with 5 mM PC for 96 hours. (D) India ink preparation of untreated H99 cells at the same magnification. Scale bars = 5 µm.

Incubations of *C. neoformans* yeast with *A. castellanii*, macrophages, and their respective extracts were evaluated in longer incubation periods for the induction of cell gigantism. At days 2, 4, and 6, co-incubation with amoeba, macrophages, and the extracts all induced larger capsule volumes when compared to incubation in PBS or minimum medium alone (Figure 7A, p<0.05). After 8 days, we observed an increase in the cell body volume of these cells and a concurrent reduction of relative capsular volume, but the overall volume of the *C. neoformans* yeast did not display a statistically significant difference. Cells incubated with amoeba extract and with the intact amoeba cell have a distinct pattern, with a double-layered capsule and two regions of different density. These cells had diameters ranging from 15 to 20 µm, approximately the size of giant cells previously described as forming *in vivo* [7], [22] (Figure 7B). Analysis by indirect immunoflourescence of capsule-induced *C. neoformans* cells after 6 days revealed stronger binding than the capsules of *C. neoformans* cells in PBS, consistent with the capsule enlargement phenomenon previously described (Figure 7C). Additionally, India ink staining and immunofluorescence of *C. neoformans* cells exposed to PC manifested very large *C. neoformans* cells as early as 48 hours that were even larger than those observed after extended incubation with *A. castellanii*, macrophages, and their respective extracts (Figure 8A, 8B, and 8C). Those cells did not constitute the majority of the cells, but were notably larger than untreated cells (Insets in Figure 8A and in 8B and Figure 8D). The whole cells averaged from 20 to 30 µm and both cell body and capsule were significantly enlarged and their relative abundance appeared to increase after longer incubation periods (Figure 8C and 8D).

## Discussion

*C. neoformans* capsular enlargement is a phenomenon that has frequently been associated with its virulence in mammals [5]. Numerous signals are known to trigger capsule enlargement including CO_2_ [29], serum [8], iron deprivation [30], pH, and certain growth conditions [22]. The fact that chemically diverse signals trigger capsule enlargement suggests that this phenomenon may be a non-specific defense against fungal-perceived stress, threats, and danger. When murine lungs are inoculated with *C. neoformans*, capsular enlargement proceeds rapidly. The phenomenon, in its extreme and combined with cellular growth, can also result in the formation of gigantic cells [31]. However, since the primary ecologic niches of *C. neoformans* are soils and trees, and animal infection may be a relatively rare event involving only a minute fraction of the cryptococcal fungal mass on the Earth, it is unlikely that capsular enlargement evolved for the specific purpose of defense in animal hosts. Given that soil amoebae have been reported to be major predators of *C. neoformans* in that niche [32], we investigated whether interactions with protozoa also induced capsular enlargement.

Incubation of *C. neoformans* with *A. castellanii* resulted in capsular enlargement. The effect required contact between the fungal and protozoan cells but did not require amoebae viability. Since the capsule protects *C. neoformans* against amoebae ingestion, and since the diameter of the capsule correlates inversely with the efficiency of phagocytosis [9], [21], capsular enlargement is a likely defense mechanism against phagocytic predators. The absence of a capsular enlargement response when cryptococci are incubated with beads implies that the stimulus is more than merely mechanical and that fungal cells discriminate between inert spheres and cells.

Since both live and dead amoebae triggered capsular enlargement and since protozoan cells represented a very different trigger than previously reported stimuli, we investigated the nature of the responsible component by fractionating amoebae cells and testing these fractions for their ability to elicit capsule growth. One of the approaches was to submit *A. castellanii* cells to lipid extraction. The upper polar phase, normally called the non-lipid fraction, had comparable efficacy to intact amoeba cells in promoting capsular enlargement. In contrast, neither interface nor the lower phase lipid extract fractions demonstrated any capsule enlargement activity. Concurrently, due to the requirement for cellular contact, we investigated the requirement for fungal phospholipase in amoebae-promoted capsular enlargement. Phospholipase B, but not phospholipase C, was required for *C. neoformans* to respond to amoebae with capsular enlargement.

Combined, these two results were conflicting since the partitioning to the upper phase during lipid extraction suggested that the enlargement activity was not due to a lipid molecule, given that most of the lipids are normally found in the lower organic phase. Additionally, the requirement for phospholipase B suggested that the molecule was a phospholipid or at least a phospholipid degradation product. The treatment with Proteinase K and the subsequent TLC analysis gave us a possible explanation for this potential inconsistency. Instead of abolishing the activity, as would be expected if the activity was due to a polypeptide chain, the treatment actually increased the extract activity and produced a new band in the TLC that was compatible with the release of a phospholipid. In the case of lipids that are covalently associated to proteins or carbohydrates, they could be carried to the non-lipid extract during phase partitioning [33]. Thus, our hypothesis is that the phospholipids responsible for the enlargement activity are strongly associated with a polypeptide and this association results in their partitioning to the upper polar fraction during the extraction. As expected, the activity was transferred to the lower phase upon Proteinase K digestion and new fractionation, supporting the hypothesis that free lipids are released. The treatment with Proteinase K disrupts this interaction, further building upon the activity of phospholipase B. Additional support for this observation comes from the fact that, even in the absence of strong interactions with other molecules, some phospholipids and other highly polar lipids, such as gangliosides, partition into the upper phase [34]. Furthermore, mass spectrometry of both intact crude amoeba polar extracts and those treated with Proteinase K indeed revealed the presence of different classes of phospholipids in our samples but their identity could not be established due to small quantities. Given that phospholipases are known to damage membranes, we interpreted this result as indicating that fungal phospholipase B catalyzed the release from the membrane of lipids and/or lipid fractions that are subsequently sensed by the fungal cells. Phospholipase B is known to be a virulence factor for *C. neoformans*, but the dependence of capsule enlargement on this activity implies a potential new role in cryptococcal biology.

Incubation with amoeba fractions also altered the production of both capsular and exopolysaccharides. We measured an approximately two-fold increase in the capsular polysaccharide and a six-fold increase in the exopolysaccharide production in the presence of amoeba lipid extracts. Given the structural differences in the capsular and exopolysaccharide fractions, the quantitative differences in production are consistent with the notion that these compounds have independent pathways of production and/or secretion [35].

The observations that protozoan phospholipids triggered capsular enlargement prompted us to investigate whether mammalian lipids had the same effect. The polar fractions extracted from macrophages and serum were also shown to trigger capsular enlargement. An interesting difference between the effects observed with amoebae and with serum was the absence of a phospholipase B requirement in the capsular enlargement response to serum-derived polar lipids. This observation suggests that serum lipids are responsible, at least in part, for the ability of serum to trigger capsule growth. However, serum also contains iron binding proteins that could conceivably indirectly trigger capsule growth through iron limitation [30].

We then attempted to identify the specific compound responsible for the capsular enlargement. Our first approach was to further fractionate the upper phase from the lipid extraction using a variety of techniques. However, activity was inevitably lost with progressive fractionation. Size exclusion and reverse phase chromatography purification of the polar fractions revealed activity in at least two fractions but mass spectrometry analysis of the most active fractions was not revealing (unpublished data). This suggested that the compound was not stable and/or that the effect required more than one molecule. Indirect evidence for the instability of the compound comes from the observation that we were never able to demonstrate capsular growth induction in experiments where fungal and amoeba cells were separated by diffusible membranes. Direct evidence for the instability of the capsule-inducing compound comes from the observation that extracts rapidly lost their activity when stored at room temperature. The instability of the activity suggests an explanation for our difficulties in the attempts to further purify and identify the active compound(s) responsible for capsule enlargement. In retrospect, the putative identification of the active compound as a phospholipid suggests an explanation for its instability since these compounds are rapidly degraded by molecular oxygen and our protocols did not involve working in oxygen-free conditions. However, it is also possible that the inability to demonstrate capsular enlargement in assays with diffusible membranes indicates strong concentration dependence such that the effect is lost with dilution.

Our second approach to molecular identification was to consider compounds that may be present in the polar extract, to obtain them in pure form, and to test them individually, and sometimes in combination, for their effects on capsule growth. Using this approach, we found that phospholipids, in particular, phosphatidylcholine (PC) and lysophosphatidylcholine (LC) and two glycerophosphodiesters, GPC and GPE, that are components of the polar head of phospholipids, were able to reproduce the *C. neoformans* capsule enlargement. Additionally, GPC and GPE were able to overcome the inability of the phospholipase B mutant to enlarge its capsule in response to the amoeba extract. This was in contrast to intact PC, supporting the necessity of PLB activity to generate these small compounds that trigger phospholipid-mediated capsule enlargement. The fact that GPC and GPE are regularly found in brain and other host tissues [36], also suggests that they could act as possible triggers for the capsule increase observed with *C. neoformans* in the host environment [37].

Although the mechanism by which phospholipids trigger capsule enlargement was not elucidated as part of this study, our working hypothesis is that certain phospholipids can trigger signaling cascades in *C. neoformans* that in term promote capsule synthesis. In this regard, we note that members of the human oxysterol binding protein (OSBP) family can bind phospholipids [38] and it is conceivable that in *C. neoformans* this highly conserved family, or another signaling set of proteins, has been specialized to bind phospholipids.

The finding that phospholipids triggered capsular enlargement led to a conundrum; neither the lower phase extract from amoebae nor from macrophages mediated this effect, however phospholipids would have been abundant in the organic layer. Our hypothesis is that the complex lipid solution in the lower phase includes both stimulators and inhibitors of capsule enlargement. In this regard, we note that this fraction would also include all the sterols and this class of compounds can trigger signal transduction by the OSBP-related protein system [39]. In yeast, stimulation of these proteins inhibits golgi vesicular production [40]. An analogous effect in *C. neoformans* could shut down capsule production since the polysaccharide is synthesized in golgi-derived vesicles [41]. In this regard we note that in the dose response data with GPC and GPE, the amount of enlargement peaked at 10 µM and declined at higher concentrations consistent with an inhibitory effect. Alternatively, there could be a possible nutritional explanation. Capsule size is known to be negatively regulated by nutrient-rich media and high glucose concentrations (reviewed in [3]). Consequently, it is possible that the lipid-rich environment of the lower phase is perceived by the fungus as nutrient-rich, leading to inhibition of capsular enlargement. Given that capsular enlargement has been associated with poor nutrient preparations and stationary phase growth conditions, combined with the recent observation that giant cell formation in *C. neoformans* follows cell cycle progression without fission [6], [7], we decided to evaluate the effects of our lipid preparations on cell growth but found no effect. Similarly, lipid-induced capsule growth was observed at all tested temperatures with the exception of extremely low, non-physiological temperatures. The occurrence of capsule enlargement at temperatures ranging from ambient to mammalian, suggests that this phenomenon can occur both in environmental niches and during mammalian infection.

We also observed that co-incubations with extracts or cells of *A. castellanii* and macrophages for extended periods induced the formation of very large cells. Although these cells did not achieve the full dimensions of giant cells described *in vivo*, they approximated that size and represented a tremendous increase in both cell and capsule sizes. Given that detailed studies of giant cells are likely to require the ability to induce them *in vitro*, the finding that these extracts promoted their formation is an important development for future progress in understanding their cell biology. This type of *C. neoformans* cells displayed a double-layered capsule, consisting of a denser region close to the cell body, and an outer layer, which permitted a higher penetration of India ink particles. Immunofluorescence studies revealed that binding of mAbs to the capsule of *C. neoformans* cells following incubation with amoeba or extracts was more intense, indicating the presence of more reactive polysaccharides surrounding the yeast. An increase in the cell body was observed after eight days of incubation with amoeba or macrophage cells and extracts when compared to PBS alone, along with a concurrent decrease in capsular volume, but no alteration in the whole cell volume. This suggests that in the conditions of starvation that accompany the late stationary phase, *C. neoformans* might be reusing the capsular polysaccharides as an energy source.

**Figure 9:**
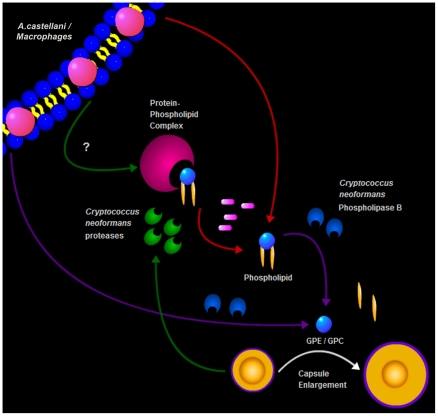
Schematic drawing summarizing the observations made in this study. *C. neoformans* is proposed to release enzymes that damage the cell membrane of amoebae and macrophages thus releasing phospholipids, possibly in combination with proteins. Those phospholipids are then cleaved by PLB, releasing their polar heads that are in turn sensed by *C. neoformans* cells, triggering capsule enlargement and the formation of giant cells.

In summary, we describe a new trigger for cryptococcal capsule enlargement that is present in the polar lipid fractions derived from amoebae, macrophages, and mammalian serum. We propose a model for *C. neoformans* capsule enlargement resulting from interactions with amoebae or macrophages, whereby *C. neoformans* induces release of phospholipids from the phagocytic cell membrane after action of PLB possibly facilitated by *C. neoformans* proteases [42]. Those phospholipids are then cleaved by PLB releasing their polar heads that are in turn sensed by *C. neoformans* cells, triggering capsule enlargement and the formation of giant cells (Figure 9). Since capsule enlargement reduces the phagocytic efficacy of both amoeba and macrophages [4], [21], we propose that this is a general cryptococcal defensive response to the sensation of potential danger. The observations with phospholipase B-sufficient and -deficient cells, suggest that fungal enzymes are used to damage amoeba cell membranes and release lipid components that subsequently trigger capsule growth. According to this view, the fungus would sense the lipid components and/or cleavage products (GPC or GPE) as signals of potential danger in the form of predatory phagocytic cells in their immediate vicinity. In this hypothesis, phospholipids join other known triggers of capsule growth such as Fe deprivation, CO_2_, and pH as stress signals to which the fungus responds by capsular enlargement. To our knowledge, these are the first host-derived compounds identified to promote capsular and cellular enlargement. Our observations provide yet another striking parallel between the response of *C. neoformans* to amoebae and macrophages. Such similarities, combined with the observations that virulence can be enhanced by passage in amoeboid cells [1], have been used to argue that the capacity for virulence in *C. neoformans* and other soil-dwelling organisms with no requirement for animal hosts is a consequence of selective pressures in soils from the presence of protozoa. Recently, the same argument has been put forward to explain the virulence of certain fungi for insects [14]. The parallel responses of *C. neoformans* to macrophages and amoebae provide additional support for the view that cryptococcal virulence is a result of selection of certain traits by environmental pressures that also enhance survival in animal hosts.

## Materials and Methods

### Organisms and culture conditions

*A. castellanii* strain 30234 and *C. neoformans* strain 24067 were obtained from the American Type Culture Collection (ATCC, Manassas, VA). The amoebae were cultured in peptone-yeast extract-glucose broth, PYG (ATCC medium 712, containing 10 mM glucose), in tissue culture flasks at 28°C. *A. castellanii* cells were used when confluent on the bottom of the flask and were passaged every 5-7days, as described [43]. The *C. neoformans* strains 24067 (serotype D) and H99 (serotype A) were grown from frozen stocks and maintained in Sabouraud dextrose broth (SDB, Difco, Lawrence, KS) or minimal medium (MM, 15 mM glucose, 10 mM MgSO_4_, 29.4 mM KH_2_PO_4_, 13 mM glycine, and 3.0 ìM thiamine). *C. neoformans* strains H99, *plb1*, and *plb1* ^REC1^ [24] were obtained from Drs. Gary Cox and John Perfect (Durham, NC). *C. neoformans* strains H99, Δ*isc1*, and Δ*isc1^REC^* strains [44] were obtained from Dr. Maurizio Del Poeta (Charleston, SC).

### Effect of amoeba co-incubation on *C. neoformans* capsule size

After growing *C. neoformans* as described above, the cells were washed 3 times in phosphate buffered saline (PBS) and 1×10^6^ yeast cells were suspended in either PBS or MM and placed in 96-, 24-, or 6-well plates. Cryptococcal cells were incubated with 1×10^6^ of either: live or dead A. *castellanii* or J774.14 macrophage-like cells. Alternatively, *C. neoformans* was incubated with 9.2 µm polystyrene beads. Incubations were done overnight, for 24 h or 48 h at 28°C. Dead amoebae were obtained by boiling the organism for 5 minutes. Lysing of amoeba cells was accomplished by forcefully pulling and pushing the cell suspension through 26.5 gauge syringe needles 15-20 times. Additionally, *C. neoformans* cells were incubated in medium conditioned by prior growth of *A. castellanii* or cell-free supernatant from a previous overnight co-incubation experiment. To evaluate the effects of temperature in the interaction, plates with *C. neoformans* strain H99 and *A. castellanii* were also incubated overnight at 4°C, room temperature, 28°C, and 37°C.

### Capsule measurement

The volume of the capsule both before and after exposure to the various conditions was measured using India ink suspensions, as previously described [8]. After overnight incubation for each condition tested, *C. neoformans* cells were washed from the wells, spun down, and in some cases stained with Uvitex 2B (Polysciences, Inc.), and then all aliquots were spotted on microscope slides, mixed with a drop of India ink, and examined using an Olympus AX70 microscope at a magnification of 40X (Center Valley, PA). Cells suspended in India ink were photographed with a QImaging Retiga 1300 digital camera using the QCapture Suite V2.46 software (QImaging, Burnaby, British Columbia, Canada). Alternatively, *C. neoformans* cells were observed in an Axiovert 200 M inverted microscope using a 40X objective (Carl Zeiss Micro Imaging, Thornwood, NY) and photographed using a Hamamatsu ORCA ERJ camera (Hamamatsu Photonics, Hamamatsu City, Japan). The volume of the *C. neoformans* capsule was measured using Adobe Photoshop 7.0 for Windows (San Jose, CA.), or AxioVision software (Carl Zeiss Micro Imaging, Thornwood, NY). The diameter of the whole cell (*D*_wc_) and the cell body (*D*_cb_) were each measured and the capsule width was defined as the difference between D_wc_ and D_cb_. diameters. The volume of the capsule was calculated using the equation for the volume of a sphere, 4/3 Π(*D*/2)^3^, such that the capsule volume (*V*_c_) was the difference between the whole cell volume (V_wc_) and the volume of the cell body (V_cb_). For each condition, we averaged the capsule volume for a minimum of 50 *C. neoformans* cells.

### Investigation into requirements for cellular contact

To determine whether cell contact was needed for *C. neoformans* to respond with capsular enlargement, experiments were carried out where fungal and amoeba cells were separated by means of filter inserts. *C. neoformans* cells were placed in 24 well plates and separated from *A. castellanii* by the presence of a cell culture insert with a 0.4 ìm pore size (BD Falcon, Franklin Lakes, NJ). Prior to co-incubation, *C. neoformans* cells were suspended in PBS and placed below the inserts. Above the inserts, either *A. castellanii* with *C. neoformans*, *A. castellanii* alone, or PBS was then placed. The plates were incubated for either 24 or 48 h at 28°C. A third group was incubated for 24 h with PBS below the filter prior to the addition of the *C. neoformans* and then *C. neoformans* was added for an additional 24 h of incubation. All organisms were suspended at a density of 1×10^6^/mL and at an initial 1∶1 ratio of fungal to amoeba cells.

### Lipid extraction

Cells from confluent *A. castellanii* cultures were collected by centrifugation at 320 x g for 10 min. J774.14 macrophage-like cells were also collected by centrifugation after growth to confluence; however, they were spun at 320 x g for 7 min. Cell pellets were washed three times with PBS. Resuspended pellets (10^8^ cells in 10 mL) of *A. castellanii*, macrophages, or aliquots of Fetal Calf Serum (FCS) were each incubated with a mixture of chloroform and methanol (2∶1 v∶v) for 2-3 h on a bench top rocker at room temperature. The samples were then centrifuged for 10 min at 1100 x g for phase partitioning and the three phases obtained (upper, interface, and lower) were collected and dried overnight in a vacuum centrifuge (Eppendorf, Hauppauge, NY, USA). Interface and upper phase lipid fractions were resuspended in PBS. Lower phase lipid fractions were resuspended in Dimethyl sulfoxide (DMSO).

### Capsular enlargement assay upon fractionation

After resuspension, the amoeba and macrophage extracts were tested in capsular enlargement activity assays with *C. neoformans* strains 24067 and H99 and serum extracts were added as well for the tests with *C. neoformans* strains H99, *plb1*, and *plb1* ^REC1^. For each activity assay, *C. neoformans* cells were washed, counted, and resuspended at 1×10^6^ cells/mL in PBS. A 1 mL volume of the cell suspension was added to each well of a 6-well plate. An additional 1 mL of PBS, and 1×10^6^ of *A. castellanii* cells, were always added to the first and second wells, respectively. In general, 1 mL of a solution of the fraction to be tested was added to each of the subsequent wells, with the concentration determined by the particular experiment. The plates were incubated at 28°C overnight, 24 or 48 h and capsule volume was measured using India ink staining (described above).

### Requirement of phospholipase activity for capsule enlargement

In addition to strains 24067 and H99, experiments were performed with *C. neoformans* strains H99, *plb1*, and *plb1* ^REC1^ to determine the effect of phospholipase B deficiency on the ability to respond to co-incubation with amoeba extracts [24]. Strains H99, Δ*isc1*, and Δ*isc1^REC^* were tested to determine the effect of phospholipase C deficiency on the ability to show activity [44].

### Stability of the capsule-inducing amoeba polar extract

The upper phase of the amoeba extract was suspended in PBS and tested for its ability to induce capsule enlargement as described above. A series of aliquots were left at room temperature and one was tested each day for the ability to elicit capsule enlargement. This experiment was conducted until no effect on capsule enlargement was observed.

### Amoeba polar extract activity following thermal and enzymatic treatments

In order to evaluate the chemical characteristics of the active molecule(s) in the polar extract, the extracts were submitted to various treatments. Treatments included: (1) heat for 1 h at 65°C, (2) Proteinase K treatment [100 µg/mL in 50 mM Tris-HCl (pH 8.0) and 1.0 mM CaCl_2_] for 1 h at 37°C followed by enzyme inactivation at 70°C for 30 min, or (3) treatment with 1 U of *Aspergillus niger* β-glucanase (Sigma Aldrich). The capsule volumes of the *C. neoformans* cells after overnight incubation with the various treated extracts were then compared to cells incubated in PBS alone or incubated with untreated extracts.

### Thin layer chromatography

To investigate what was released after the enzymatic treatments listed above, thin layer chromatography (TLC) was performed. A similar volume of all the fractions to be tested was dried, resuspended in chloroform, and 25 µL were spotted onto the membrane. A general separation of phospholipids based on head group polarity was done using a mobile phase composed of chloroform∶methanol∶water (65∶25∶4). Four of the main phospholipids known to be present in A. castellanii cells, L-α-Phosphatidylethanolamine (unsaturated, from Glycine max), L-α-Phosphatidylcholine (unsaturated, from Glycine max), L-α-Phosphatidylinositol (unsaturated, from Glycine max), and 1,2-Diacyl-sn-glycero-3-phospho-L-serine (unsaturated, from bovine brain) were purchased from Sigma-Aldrich (St. Louis, MO) [45] and used as standards. TLC plates were dried and stained in an iodine vapor chamber until the spots formed.

### Fractionated extracts tested upon enzymatic treatment

Upon treatment with Proteinase K as described above, samples were submitted to a second round of fractionation with a mixture of chloroform and methanol (2∶1 v∶v) for 2–3 h as described above. Upper and lower phases were then tested for capsular enlargement activity as described above.

### Purified molecules tested for capsular enlargement activity

Phospholipids known to be present in *A. castellanii* were purchased from Sigma-Aldrich (St. Louis, MO) [45]. 3-sn-Phosphatidic acid sodium salt from egg yolk lecithin (PA), L-α-Phosphatidylcholine from egg yolk (PC), L-α-Phosphatidylethanolamine from egg yolk (PE), L-α-Phosphatidyl-DL-glycerol sodium salt from egg yolk lecithin (PG), L-α-Phosphatidylinositol from Glycine max (PG), 1,2-Diacyl-sn-glycero-3-phospho-L-serine from bovine brain (PS), and L-α-Lysophosphatidylcholine from bovine brain (LC), were each tested with *C. neoformans* for their ability to induce capsular enlargement. Arachidonic Acid, Epoxyeicosatrienoic Acid, Thromboxane B2, Prostaglandin E2, Prostaglandin I2, Leukotriene B4, and Leukotriene C4 were also purchased from Sigma-Aldrich (St. Louis, MO). The powders were resuspended in PBS, MM, or ethanol, serially diluted, and tested in the activity assay with *C. neoformans* strains H99 and 24067. Purified glycerophospholethanolamine (GPE) and glycerophosphocholine (GPC) were purchased from Avanti Polar Lipids (Alabaster, Alabama). These two substances were tested in activity assays with *C. neoformans* strains 24067, H99, *plb1*, and *plb1* ^REC1^. For GPC and GPE, we tested concentrations ranging from 0.1 µM to 1 mM and found that 10 µM was the lowest concentration where we observed activity. The activity did not increase at higher concentrations. For PC, we chose a 5 mM concentration based previous studies [26]. Additionally, we carried out a dose response study and found that for PC the effect was higher at concentrations equal to or higher than 1 mM. As the effects of GPC, GPE, and PC were stronger in MM in comparison to PBS, most of the tests were done in this condition.

### Exopolysaccharide and capsular polysaccharide production under treatment with amoeba extract

*C. neoformans* cells were incubated with amoeba extracts in either PBS or MM and the resulting pool of polysaccharide was evaluated by ELISA. Exopolysaccharide and capsular polysaccharide were measured by an inhibition ELISA on reaction plates to which 10 µg/well of glucuronoxylomannan (GXM) [46] was affixed overnight, followed by blocking with 1% (w/v) bovine serum albumin diluted in PBS (blocking buffer), and then subjected to mAb binding [47]. A second blank ELISA plate (inhibition plate) was blocked for 1 h at 37°C and a solution of 2 µg/mL of mAb 18B7 [48] was incubated with serial dilutions of GXM (concentrations of 10 µg/mL to 0.06 ng/mL) or capsular and exopolysaccharide samples at 37°C for 1 h. Contents of the wells were transferred to blocked reaction plates with adherent GXM as antigens. After incubation at 37°C for 1 h, the plates were washed and anti-mouse IgG conjugated with alkaline phosphatase (1∶1000 in blocking buffer) was added to the wells for 1 h at 37°C. The plates were again washed, incubated with a *p*-nitrophenyl phosphate substrate solution and read at 405 nm. The concentration of GXM in the samples was calculated extrapolating from the GXM standard curve.

### Immunofluorescence

Aliquots of *C. neoformans* suspensions following incubation with amoeba extracts were washed with PBS, centrifuged, and suspended in a 50 µg/mL solution of 18B7 mAb in a 5% Bovine serum albumin solution in PBS. Tubes were incubated for 1 h at 37°C while shaking and then washed three times with PBS. The pellets were suspended in 100 µl of a 1∶100 dilution of FITC-conjugated goat anti-mouse IgG1 (Southern Biotechnology) in blocking solution. Tubes were again incubated for 1 h at 37°C and washed with PBS. Pellets were then suspended in mounting medium (Biomeda Corp, Foster City, CA) and spotted onto a microscopy slide. Slides were examined with an Olympus AX70 fluorescence microscope using a 495 nm filter, at a magnification of 40X. Alternatively, 10^7^ *C. neoformans* cells were stained for 1 h with 10 µg/mL DTAF-labeled 18B7 mAb and 0.01% Uvitex 2B. After washing, cells were suspended in Prolong gold anti-fade mounting medium (Invitrogen, Carlsbad, CA) and imaged using a 63x 1.4 objective. Z-stacks were collected and deconvolved using a constrained iterative algorithm from AxioVision software (Carl Zeiss Micro Imaging, Thornwood, NY).

### Growth Curves

A Bioscreen-C Automated Growth Curve Analysis System (Growth Curves USA, Piscataway, NJ) was used to measure the growth of *C. neoformans* strain 24067 in the presence of the *A. castellanii* polar extract. The fungal cells were incubated either alone in PBS or in PBS and 1 µL or 10 µL of polar extract and compared with the cells incubated in SDB alone or in SDB and 1 µL or of 10 µL polar extract.

### Mass spectrometry of polar extracts

Polar extracts and polar extract samples after treatment with proteinase K and lipid re-extraction by the FoIch method were submitted to mass spectrometry analysis by the Kansas Lipidomics Research Center (Manhattan, KS).

### Statistical analysis

Statistical analysis was performed using GraphPad Prism 5 (La Jolla, CA). The Shapiro-Wilk test was used to verify normal distribution of the measurements. To determine significance, One-way ANOVA tests were used, followed by either correction with the Bonferroni test for multiple pairwise comparisons for normally distributed values or the Kruskal-Wallis analysis for measurements that were not normally distributed. *P* values of less than 0.05 were considered significant.

## Supporting Information

Figure S1 A. Dose-response effects of phosphatidylcholine (PC) on capsule enlargement of *C. neoformans*. B. Effects of different phospholipid classes on capsule enlargement of *C. neoformans*. *C. neoformans* cells were incubated for 48 h in minimal media (MM) alone or MM containing 1 mM of one of the following lipids: phosphatidic acid (PA), phosphatidylcholine (PC), phosphatidylethanolamine (PE), phosphatidylglycerol (PG), phosphatidylinositol (PI), phosphatidylserine (PS), or lysophosphatidylcholine (LC). All the compounds, with the exception of PG and PS, produced significant enlargement of the *C. neoformans* capsule volume. PC and LC also produced significant enlargement of *C. neoformans* cell body volume. Conversely, PS produced significantly smaller cells. (*) denote p<0.05 relative to cells in minimal media alone.
(TIF)

Figure S2 Dose response effects of GPC, GPE on capsule enlargement of *C. neoformans*. *C. neoformans* cells were incubated for 48 h in minimal media (MM) alone or MM containing different concentrations of GPC and GPE. (*) denotes p<0.05 relative to cells in minimal media alone.
(TIF)

Figure S3 Effects of PLB inhibitors on *C. neoformans* capsule enlargement induced by phosphatidylcholine (PC). *C. neoformans* 24067 cells were incubated for 48 h in minimal media (MM) alone or in MM containing 5 mM PC alone or 5 mM PC with compound AX, compound O, or palmitoyl carnitine (PAC). Compound AX, which targets mainly secreted PLB activity, did not affect the capsule enlargement, while compounds O and PAC abolished the effects of PC. (*) denote p<0.05 relative to cells in minimal media alone.
(TIF)

Table S1 List of compounds that failed to elicit capsular enlargement of *Cryptococcus neoformans* cells suspended in PBS.
(TIF)
