# Supplementary figures and images for: Phospholipids Trigger Cryptococcus neoformans Capsular Enlargement during Interactions with Amoebae and Macrophages
Source: PLoS Pathog. 2011 May 26;7(5):e1002047. doi: 10.1371/journal.ppat.1002047 (PMC3102711; doi:10.1371/journal.ppat.1002047)

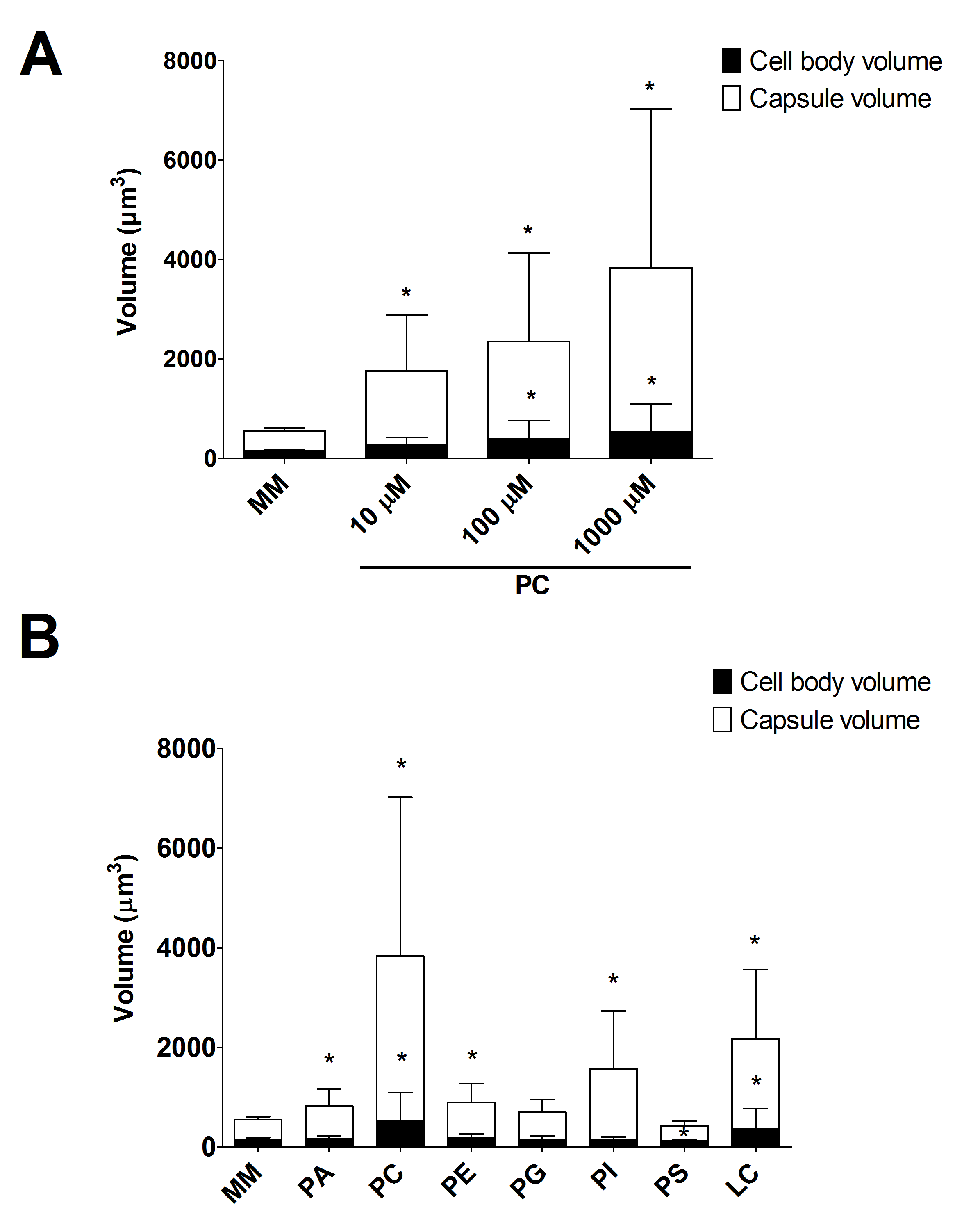

Supplement: Figure S1 — A. Dose-response effects of phosphatidylcholine (PC) on capsule enlargement of C. neoformans. B. Effects of different phospholipid classes on capsule enlargement of C. neoformans. C. neoformans cells were incubated for 48 h in minimal media (MM) alone or MM containing 1 mM of one of the following lipids: phosphatidic acid (PA), phosphatidylcholine (PC), phosphatidylethanolamine (PE), phosphatidylglycerol (PG), phosphatidylinositol (PI), phosphatidylserine (PS), or lysophosphatidylcholine (LC). All the compounds, with the exception of PG and PS, produced significant enlargement of the C. neoformans capsule volume. PC and LC also produced significant enlargement of C. neoformans cell body volume. Conversely, PS produced significantly smaller cells. (*) denote p<0.05 relative to cells in minimal media alone. (TIF) [file ppat.1002047.s001.tif]

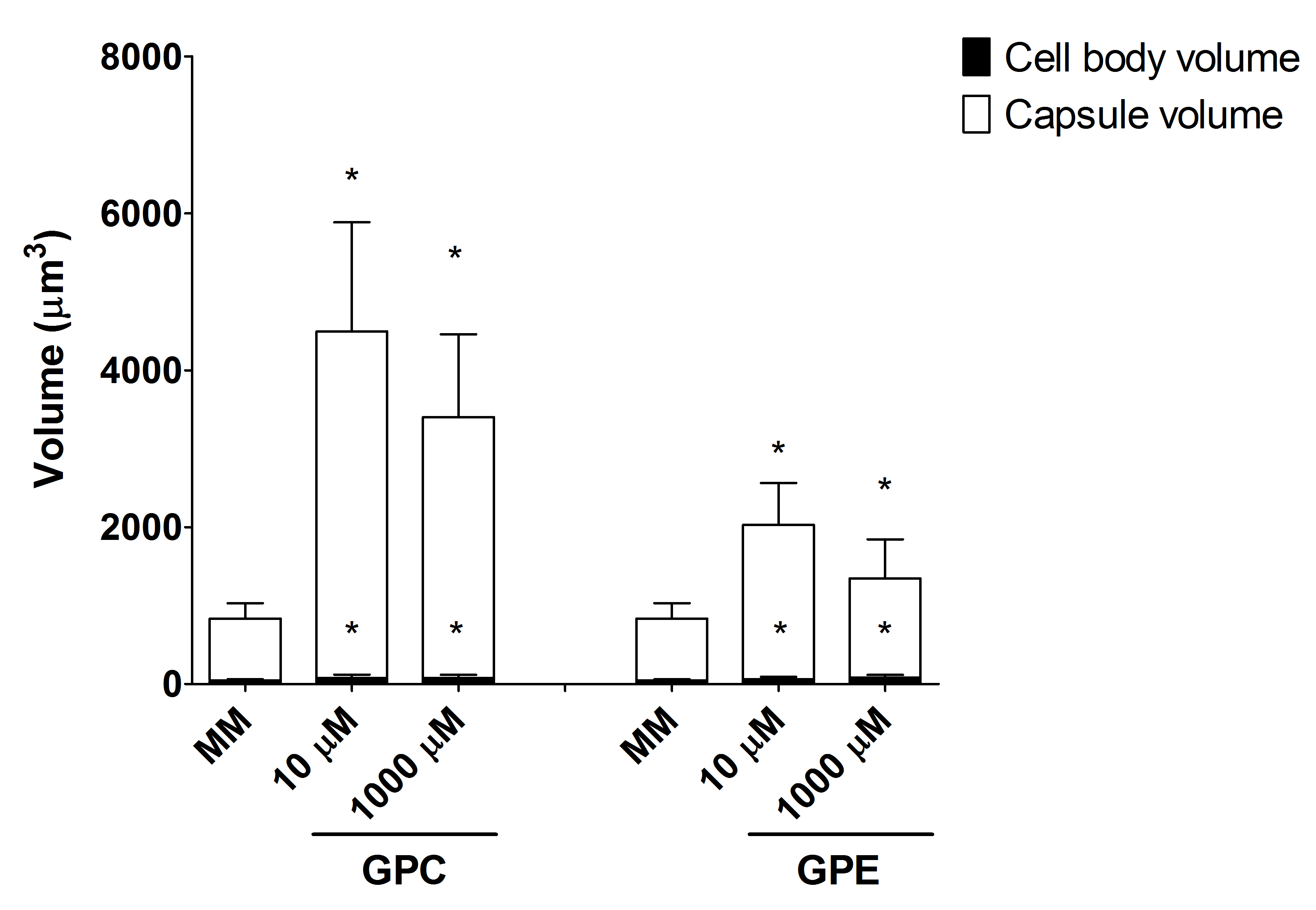

Supplement: Figure S2 — Dose response effects of GPC, GPE on capsule enlargement of C. neoformans. C. neoformans cells were incubated for 48 h in minimal media (MM) alone or MM containing different concentrations of GPC and GPE. (*) denotes p<0.05 relative to cells in minimal media alone. (TIF) [file ppat.1002047.s002.tif]

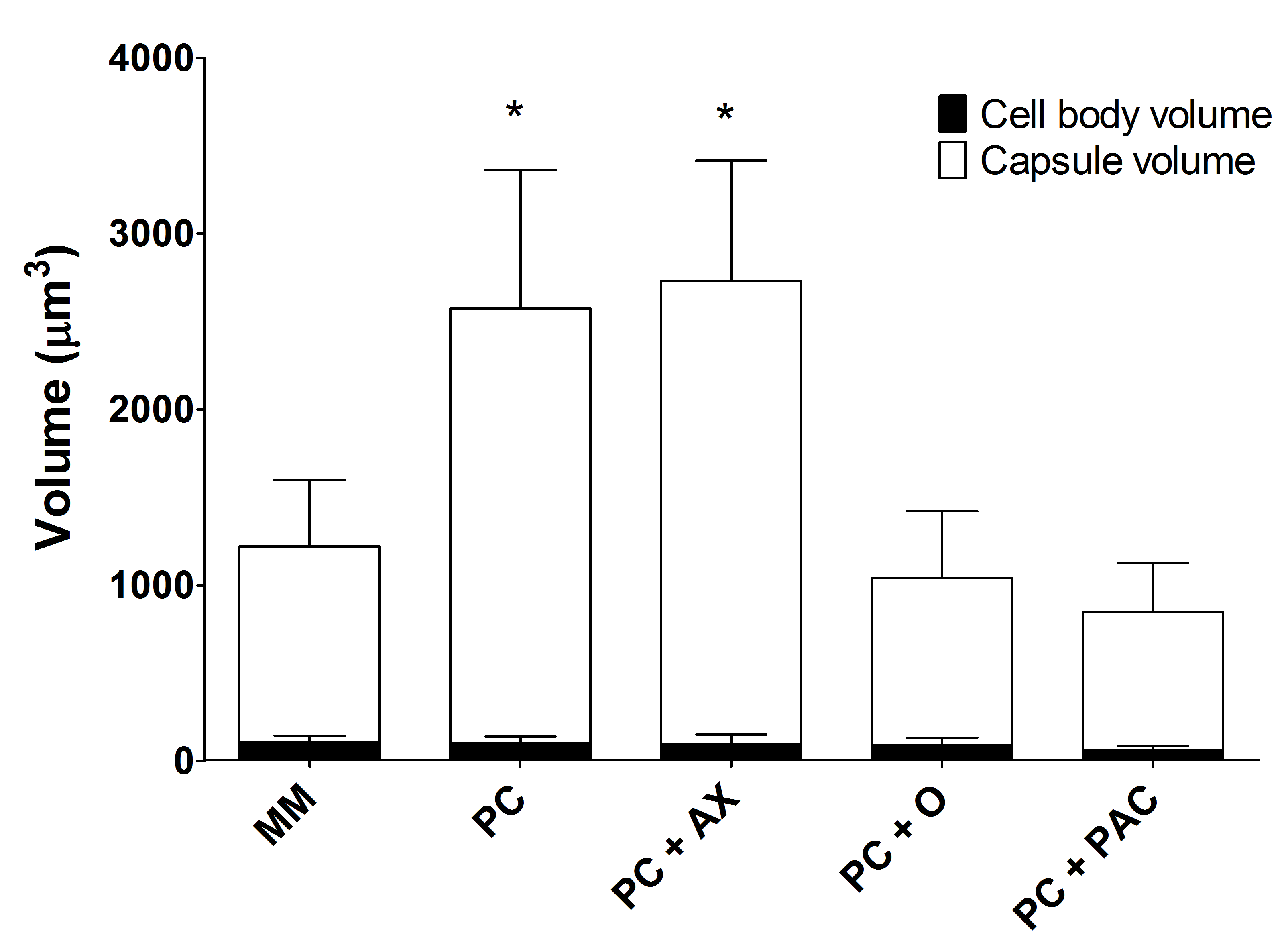

Supplement: Figure S3 — Effects of PLB inhibitors on C. neoformans capsule enlargement induced by phosphatidylcholine (PC). C. neoformans 24067 cells were incubated for 48 h in minimal media (MM) alone or in MM containing 5 mM PC alone or 5 mM PC with compound AX, compound O, or palmitoyl carnitine (PAC). Compound AX, which targets mainly secreted PLB activity, did not affect the capsule enlargement, while compounds O and PAC abolished the effects of PC. (*) denote p<0.05 relative to cells in minimal media alone. (TIF) [file ppat.1002047.s003.tif]

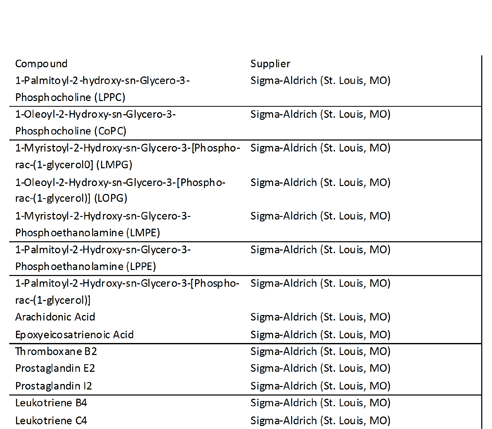

Supplement: Table S1 — List of compounds that failed to elicit capsular enlargement of Cryptococcus neoformans cells suspended in PBS. (TIF) [file ppat.1002047.s004.tif]
